# Resting-state functional magnetic resonance imaging reveals brain remodeling after Tuina therapy in neuropathic pain model

**DOI:** 10.3389/fnmol.2023.1231374

**Published:** 2023-07-12

**Authors:** Zhiwei Wu, Guangxin Guo, Yuwen Zhang, Yunyi Li, Tianxiang He, Qingguang Zhu, Lingjun Kong, Min Fang

**Affiliations:** ^1^Yueyang Hospital of Integrated Traditional Chinese and Western Medicine, Shanghai University of Traditional Chinese Medicine, Shanghai, China; ^2^Institute of Tuina, Shanghai Institute of Traditional Chinese Medicine, Shanghai, China; ^3^Department of Acupuncture and Tuina, Shanghai University of Traditional Chinese Medicine, Shanghai, China; ^4^Institute of Science and Technology for Brain-Inspired Intelligence, Fudan University, Shanghai, China; ^5^Shuguang Hospital, Shanghai University of Traditional Chinese Medicine, Shanghai, China

**Keywords:** Tuina, neuropathic pain, cortical plasticity, functional magnetic resonance imaging, amplitude of low frequency fluctuation

## Abstract

Tuina, a method of traditional Chinese manual manipulation, is an effective alternative therapy for neuropathic pain (NP), but its analgesic mechanism remains unclear. In this study, we used resting-state functional magnetic resonance imaging (R-fMRI) to explore the analgesic mechanism of Tuina in an NP rat model. After undergoing surgery to induce chronic compression of the dorsal root ganglion (CCD), one group of rats underwent Tuina at the ipsilateral BL40 acupoint once a day for 10 min during the 25 days following surgery while another group did not. Behavioral tests were performed at baseline, on the third day following surgery, and once a week for the next 4 weeks. R-fMRI was performed at baseline and 7 days and 28 days following surgery. Behavioral testing revealed that the Tuina group presented a significant response improvement to mechanical and thermal nociception stimuli compared to the untreated group 2 weeks following CCD surgery. Interestingly, rats submitted to Tuina presented higher measures of spontaneous neuronal activity in basal forebrain region, primary somatosensory cortex barrel field, dentate gyrus, secondary somatosensory cortex, striatum, descending corticofugal pathways, and globus pallidum of the left hemisphere 4 weeks after the CCD surgery compared to rats having undergone CCD only. In addition, on the 28th day, the ALFF signals of the left dentate gyrus, left secondary somatosensory cortex, left striatum, and bilateral primary cingulate cortex were significantly increased while those in the right dentate gyrus and bilateral periaqueductal gray were significantly decreased compared to those on the 7th day. Correlation analysis showed that the ALFF values of the left descending corticofugal pathways and globus pallidum had a positive correlation with mechanical withdrawal threshold and paw withdrawal thermal latency tests. Altogether, these results indicate that NPP induced by CCD surgery affects the plasticity of the cerebral cortex, and that Tuina alleviate pain behavior by promoting cortical remodeling.

## 1. Introduction

Neuropathic pain (NP) is commonly characterized as pain perception arising from nervous system impairment, often caused by central, or peripheral nervous system damage or disease, metabolic disorders, infection, neurotoxicity of drugs or radiotherapy, or infarction (stroke) (Colloca et al., 2017). Spontaneous pain and hyperalgesia are the main clinical features of NP (Jensen and Finnerup, 2014). The sensory experiences of pain that patients describe are commonly denoted as electric shock, pricking, firing, burning, numbness, or throbbing (Finnerup et al., 2016). In addition, neuropathic pain may cause emotional reactions including sleep disorders, depression, and anxiety (Finnerup et al., 2021). Epidemiological research shows that NPP impacts approximately 7–8% of the global population (Murnion, 2018), but its prevalence varies from region to region, with a high prevalence in China (17.7%) (Han et al., 2020) with a high prevalence in the United States (10–20%) (Van Hecke et al., 2014; Boehnke et al., 2016), and an average prevalence in Europe (6.9–8.2%) (Bouhassira et al., 2008). Due to its high incidence, painful nature, long duration, social and economic burden, and ability to damage to the patient’s physical and mental health, NP has attracted significant research interest in the past few decades (Bielewicz et al., 2023).

Tuina, a traditional Chinese method of manual manipulation, which has been used in China for thousands of years and guided by the theory of Chinese medicine, is an external treatment for various conditions, including but not limited to chronic pain, neurodegenerative diseases, cancer, immune diseases, sleep disorders and skin diseases (Field, 2016). In practice, Tuina therapy exerts its effects on the certain parts or points of the body, including but not limited to the head, face, trunk, and limbs, through a range of manual manipulations, including kneading, rubbing, and pressing, to maintain homeostasis of physiological and pathological conditions (Guo et al., 2016; Wang et al., 2022). Owing to its remarkable therapeutic effect, affordability, simple operation, and lack of toxic side effects, Tuina therapy is used as an alternative and complementary treatment in the treatment of pain (Liu et al., 2021). Clinical investigations have shown that Tuina therapy has demonstrated efficacy in managing different types of pain (Dhanani et al., 2011; Dabbour et al., 2018), and is most widely used in spinal diseases (Cao et al., 2022; Cheng et al., 2022). NP is common among individuals with spinal disorders, with a prevalence rate ranging from 36 to 55% of individuals (El Sissi et al., 2010; Yamashita et al., 2014). Research has confirmed that Tuina has effects on the inflammation pathway, ion channels, and glial cells, resulting in functional adaptations of the brain in individuals with peripheral nerve injuries (Liu et al., 2022).

Functional magnetic resonance imaging (fMRI), a non-invasive neuroimaging modality, is one of the most advanced technologies utilized for assessing brain activity (Savallampi et al., 2023). Its basic principle is to demonstrate the level of neuronal stimulation and functional connectivity of brain regions by detecting a change in the ratio of local oxyhemoglobin to deoxyhemoglobin. The advantage of fMRI is that it can monitor ongoing brain activity in real-time, thereby studying the synergistic effects of different brain regions during specific tasks (Torrecuso et al., 2023). Moreover, fMRI can provide high-resolution images that display the activity of different brain regions and can use different data analysis methods to explore topological configuration and brain network functions (Massalha et al., 2023). Notably, it has been shown that fMRI can detect the impact of different types and intensities of pain on brain activity and indicate the effect of pain on activation of multiple brain regions (Wager et al., 2013). Hence, fMRI has become a common practice to investigate the impact of specific treatment methods on pain-related brain circuitry (Yu et al., 2022; Savallampi et al., 2023).

Non-pharmacological therapies such as Tuina and acupuncture are considered an effective alternative pain treatment (Liu et al., 2021; Zhang et al., 2021). Brain fMRI has been used to explore the central regulatory mechanisms of non-pharmacological therapies in pain relief. For example, one resting-state fMRI (R-fMRI) experiment following nerve injury showed that electroacupuncture induces changes of both activation level of brain regions and functional connections between brain regions (Wu et al., 2018), and fMRI observations in chronic shoulder pain patients treated with ipsilateral or contralateral acupuncture revealed variations in the degree centrality (Yan et al., 2020). However, only a few fMRI studies have focused on the mechanism of the analgesic effects of Tuina.

In the present study, we evaluated intrinsic neuronal activity level upon Tuina manipulation *via* R-fMRI as measured by amplitude of low frequency fluctuation (ALFF), which is correlated to basal brain metabolism and neuromodulation (Zuo et al., 2010b). ALFF has been extensively used in pain research to investigate the effects of pain on neuronal activity and functional states in different brain regions, and to evaluate the regulatory effects of different therapies (Wu et al., 2018; Xing et al., 2021). In particular, it has been shown that acute pain result in ALFF elevation throughout the brain regions associated with pain, including the dorsal anterior cingulate gyrus, the lower hippocampus, and the dorsal thalamus (Huang et al., 2012). Meanwhile, long-term pain leads to an ALFF reduction and affects the activity of brain regions (Wu et al., 2018; Xing et al., 2021). In addition, recent studies have used fMRI to decipher the effects of non-pharmacological therapies and have observed changes in ALFF in multiple regions of the brain, representing reduced pain perception (Jiang et al., 2015; Wu et al., 2019). One study demonstrated higher ALFF within the sensorimotor cortex opposite to the impaired limb after Tuina therapy in a sciatic nerve transection model, suggesting that Tuina therapy contributes to the promotion of adaptive neuroplasticity within the somatosensory cortex and ultimately results in peripheral nerve function restoration following injury and subsequent repair (Xing et al., 2021). In the current investigation, we established a rat model of NP that was subjected to 25 days of Tuina intervention and conducted R-fMRI to explore the changes in cortical plasticity resulting from the prolonged impact of Tuina therapy. With this study, we aimed to gather new evidence of enduring effects of Tuina on resting-state cortical plasticity in individuals with NP.

## 2. Materials and methods

### 2.1. Animals

The current investigation was granted approval by the Ethics Committee of Yueyang Hospital of Integrated Traditional Chinese and Western Medicine, affiliated with Shanghai University of Traditional Chinese Medicine, on October 18, 2022 (approval No. YYLAC-2022-148-5). All procedures were conducted according to the Guide for the Care and Use of Laboratory Animals as outlined by the US National Institutes of Health and adhered to the Animal Research Reporting standards: *In vivo* Experiments (ARRIVE) guidelines. A total of 30 male Sprague–Dawley rats, aged between 7 to 8 weeks and weighing between 180 to 200 g, were purchased from Shanghai Jihui Experimental Animal Breeding Co., Ltd. (Shanghai, China; license No. SCXK (Hu) 2017–0012). Three to four rats were housed in each cage and provided with libitum water and food, in a regulated laboratory environment: 22°C temperature, constant humidity (40%) and 12 h/12 h light/dark cycle.

### 2.2. Groups and CCD surgery

Rats were randomly separated into three groups. The sham operation group (Sham) underwent skin incision and muscle separation but no further insertion. Animals in the chronic compression of the dorsal root ganglion group (CCD) only had the CCD surgery; while Animals in the Tuina group (Tuina) underwent CCD surgery followed by Tuina intervention. On the day of surgery (D0), the rats were anesthetized with an intraperitoneal injection of pentobarbital sodium (Sigma-Aldrich, St. Louis, MO, United States) at a dose of 40 mg/kg and maintained at a temperature between 36.5 and 37.5°C using an electric blanket equipped with rectal temperature monitoring capabilities for surgery. Animals designated for CCD and Tuina groups were subjected to CCD surgery as previously described (Yao et al., 2022). Briefly, a L-shaped rod with a 5 mm long-side length, a 3 mm short-side length and 0.4 mm diameter was inserted into the right intervertebral foramen of L3 and L4. As MRI instrument are made of puissant magnets, and to ensure animal safety, these rods were made of a non-magnetic titanium alloy. After surgery, the correct placement of the rod was verified through x-ray imaging (Samsung XGEO GU60, Seoul, South Korea).

### 2.3. Tuina intervention

**Figure 1:**
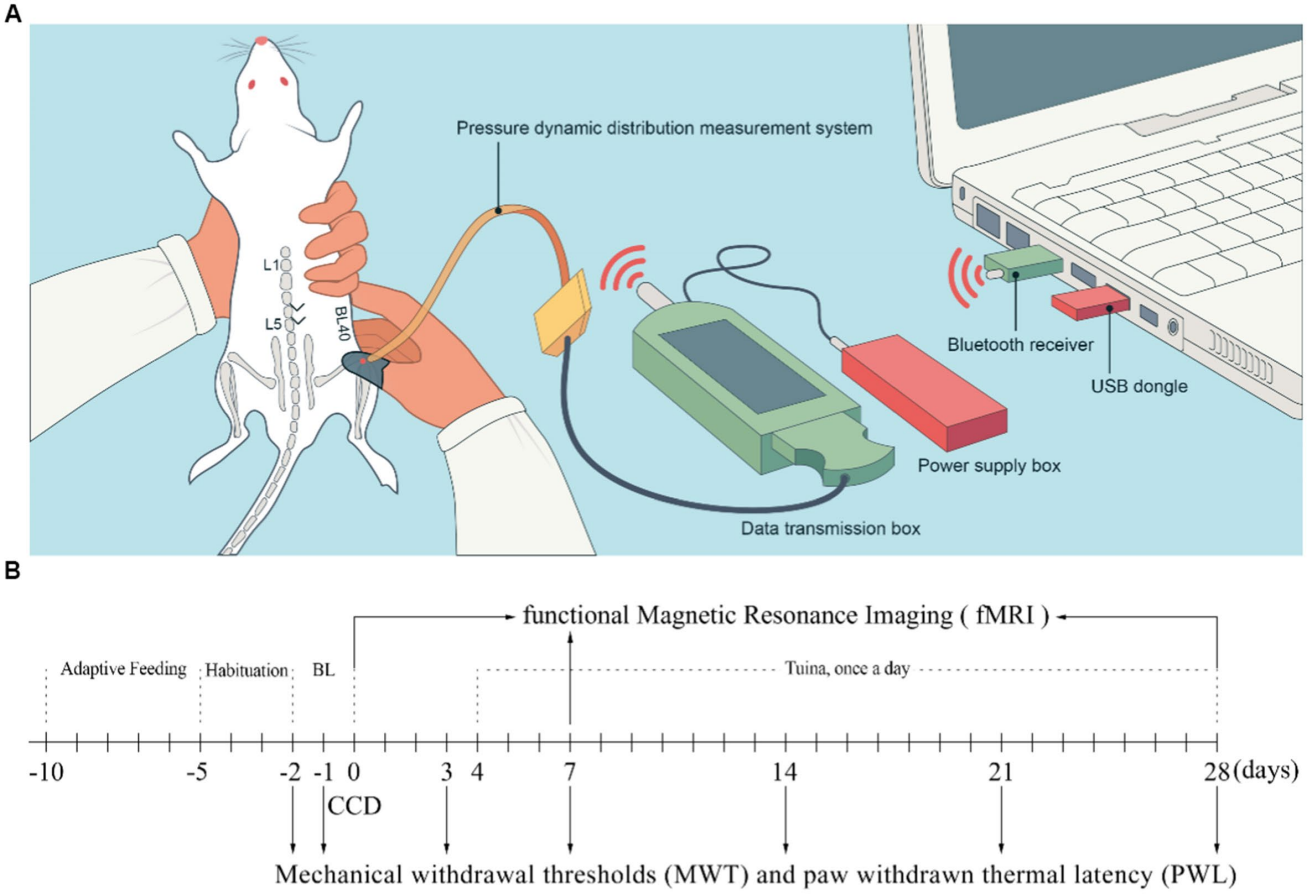
Schematic diagram of Tuina therapy and experimental flowchart. **(A)** Schematic diagram of Tuina therapy. **(B)** Experimental flowchart. BL, Baseline; CCD, Chronic compression of the dorsal root ganglion.

On the fourth day (D4) after CCD surgery, rats in the Tuina group were placed in a fixator at prone position with hind limb fully exposed for Tuina. Beforehand, habituation was carried out by placing the animals in the fixator for 10 min per day for 3 consecutive days to adapt to the environment. Tuina treatment was performed on the ipsilateral acupoint Weizhong (BL40), with thumb pressing and rubbing for 10 min once a day for 25 days (Figure 1A). To standardize the manipulation, a tactile pressure recording instrument (Novel Pliance-X 32 Expert, Germany) was used to ensure a stimulation pressure of 5 ± 0.5 N and a frequency of 2 Hz (Yao et al., 2022). To avoid any bias due to rat handling, animals from the sham and CCD groups were also habituated and placed on the fixator for the same amount of time as Tuina rats, but did not receive Tuina treatment.

### 2.4. Behavioral tests

Paw withdrawal thermal latency (PWL) and mechanical withdrawal threshold (MWT) are the commonly used methods to test mechanical allodynia and thermal hyperalgesia in experimental animals (Yao et al., 2022). Behavioral tests were conducted by two investigators blind to the groups from 08:00 to 18:00. Before the baseline test day, rats were exposed to the test environment, a 20 × 10 × 20 cm plexiglass cage, for 2 h per day on 3 consecutive days, as well as for 30 min before each test to let them adapt. Each baseline test was conducted on D-2 and D-1 and the result was determined by computing the mean value of the two tests. The behavioral tests were subsequently conducted 5 times per day on D-2, D-1, D3, D7, D14 and D28 following the surgery (Figure 1B). The central component of the right hind paw was the target stimulation point in both MWT and PWL tests. Paw withdrawal, flinching, or licking was regarded as favorable indication (He et al., 2019). Minimum and maximum values were excluded from the 5 raw values obtained at each time point, and the other 3 values were averaged as the final outcome. The electric von Frey aesthesiometer (ALMEMO 2450, IITC/Life Science, Woodland Hills, CA, United States) was utilized to conduct measurement of MWT at intervals exceeding 1 min (Yao et al., 2022). PWL was measured by a radiant heat device (Model 390, IITC Life Science) while rats were housed on a transparent double-glazing platform fitted with an electric heating wire at a temperature of 65°C (Hargreaves et al., 1988) and a cutoff time of 20 s. To prevent sensitization caused by radiant heat, the interval between stimulations was set to 10 min.

### 2.5. fMRI image acquisition

Brain fMRI scans were conducted with a Bruker 11.7 T MRI system (BioSpec117/16 USR-TT, Bruker, Karlsruhe, Germany) with a 16.0 cm aperture and a 12 cm gradient coil before surgery (baseline) and at D7 and D28 following CCD surgery. The radiofrequency (RF) coil contained a 72 mm inner diameter volumetric coil as the excitation coil and a four-channel surface coil as the receiving coil. After anesthesia with 4% isoflurane (RWD Life Science Co., Ltd., Shenzhen, China), the rats were attached to the scanner in supine position, with the head fixed to the rat specific coil and placed at the center of the magnetic field. The automatic heating pad maintained the anal temperature of rats between 36°C and 38°C. Continuous anesthesia was maintained with ventilated dexmedetomidine hydrochloride (Sigma Aldrich) under continuous respiratory monitoring. An interleaved single echo planar imaging sequence adhered to the following parameters: flip angle: 90°; slice thickness: 0.5 mm; repetition time: 2000 ms; echo time: 12.8 ms; mean value: 1; field of view: 30 × 30 mm^2^ with 128 × 128.

### 2.6. fMRI data preprocessing

The fMRI data was preprocessed *via* the Statistical Parametric Mapping 12 (SPM 12) toolbox,^1^ which relies on the MATLAB 2017a platform (MathWorks, Natick, MA, United States). Firstly, format conversion, and noise processing were performed. Raw data were converted from Digital Imaging and Communications in Medicine format to Neuroimaging Informatics Technology Initiative format and the images underwent up to 10 × 10 × 10 times augmentation to meet human brain size without requiring interpolation, enhancing the processing algorithms originally developed for human data. Non-brain tissue was eliminated through a semi-manual process prior to additional preprocessing. The fMRI images underwent slice timing procedures to prevent timing bias arising from slice acquisition. A transformation of the rigid body type was utilized to achieve spatial realignment of the images, hence rectifying voxel misalignment that had arisen from head motion. Finally, a two-step registration method was employed to achieve standard spatial normalization after head movement to the Montreal Neurology Institute space, and the data were resampled to be adjusted to a voxel size of 3 × 3 × 3 mm. We selected 27 voxel smoothing kernels (6 mm) for spatial smoothing to reduce the impact of decreased frequency of linear drive and increased frequency of physical smoothing and noise.

### 2.7. Amplitude of low frequency fluctuation analysis

Regression out of covariates was first performed using linear regression to remove confounding variables, including six mixed variables of head movement indicators and their first-order time derivatives, cerebrospinal fluid signals, and white matter signals. The bandpass filtering of the dataset was performed with a range of 0.01 to 0.08 Hz, preserving the signal with physiological significance. The application of the fast Fourier transform algorithm enabled the transformation of time series data pertaining to individual voxels from the time domain to the frequency domain. The power spectrum within the frequency of each voxel range was acquired. Considering that the frequency component amplitude determines the power at that frequency, which is in direct proportion to the square of its amplitude, the power spectrum was subjected to a square root transformation for every frequency. Subsequently, the mean square root (the ALFF value) of the power across various frequency bands was computed (Yang et al., 2007).

### 2.8. Statistical analysis

Although there was no use of statistical methods for predetermining the required number of animals per group in our study, the sample sizes utilized were comparable to those documented in a prior publication (Xing et al., 2021). Behavioral analyses were conducted by researchers blinded to the groups, while the examination, and analysis of MRI was conducted without blinding to experimental status. Analysis was performed with SPSS 21.0 (IBM Inc., Chicago, IL, United States). The outcomes of behavioral tests are noted as the mean ± standard error of the mean. The behavioral data were analyzed with a two-way repeated measure analysis of variance (ANOVA), followed by Bonferroni’s multiple comparison tests. Where data did not exhibit normality or homoscedasticity, a non-parametric test (Kruskal–Wallis k samples) was conducted. Analysis of ALFF was performed by the DPABI statistical toolbox from the SPM12 software provided in MATLAB 2017a (Yan et al., 2016). To observe longitudinal differences in ALFF, we conducted a dual sample t test on ALFF plots at different times within the groups [*p* < 0.001 Gaussian random field (GRF), GRF corrected, voxel-p < 0.001, cluster-*p* < 0.01, double tailed], indicating a statistical difference. We used one-way ANOVA to examine the differences in ALFF between baseline and post-CCD surgery D7 and D28 in the Tuina and CCD groups. Spearman correlation coefficients between ALFF values and behavioral tests were calculated utilizing SPSS 21.0 (IBM Inc., Chicago, IL, USA) and the threshold for significance was set at *p* < 0.05.

## 3. Results

### 3.1. Tuina ameliorates both mechanical and thermal allodynia induced by CCD

**Figure 2:**
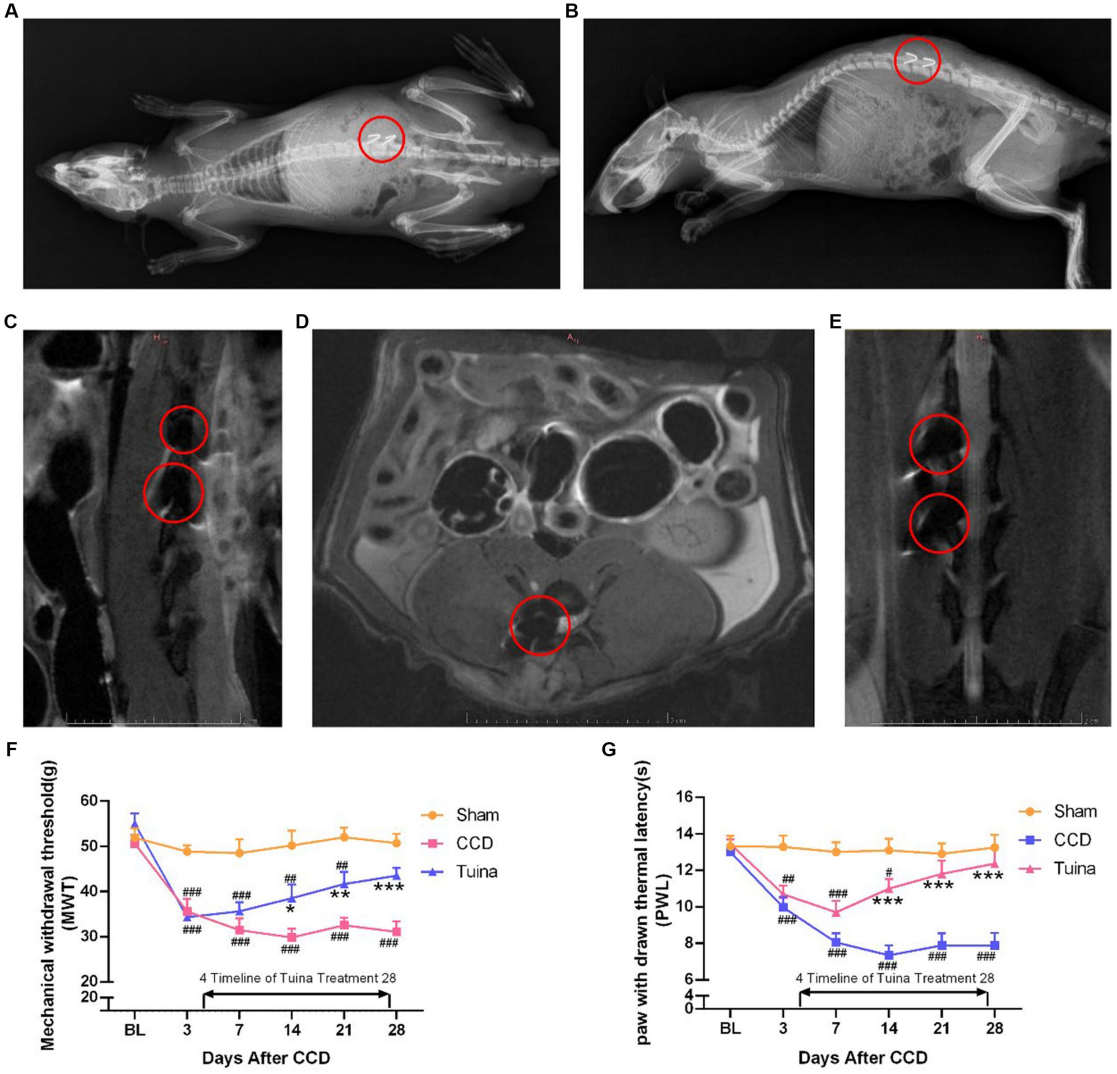
Effects of Tuina on behavior test in CCD rat model. **(A)** Coronal view of X-ray imaging in CCD rat model. **(B)** Sagittal view of X-ray imaging in CCD rat model. **(C)** Sagittal view of magnetic resonance imaging in CCD rat model. **(D)** Horizontal view of magnetic resonance imaging in CCD rat model. **(E)** Coronal view of magnetic resonance imaging in CCD rat model. **(F,G)** MWT and PWL of rats in three groups. All data are expressed as mean ± SEM (n = 9 in the Sham and CCD group and *n* = 10 in the Tuina group). Repeated measurement ANOVA was used to analyze the data at various time points between the groups and one-way ANOVA followed by LSD *post hoc* analysis was used for pairwise comparison. *p < 0.05, **p < 0.01, ***p < 0.001 vs. the CCD group; ^#^*p* < 0.05, ^##^*p* < 0.01, ^###^*p* < 0.001 vs. the Sham group. MWT, Mechanical withdrawal threshold; PWL, paw withdrawal thermal latency; SEM, Standard Error of Mean.

To determine the mechanism by which Tuina, used as a non-invasive pain-relief therapy for centuries (ref), exerts its analgesic effects, we first looked at its effects on pain sensitivity. For this, we used a CCD rat model that we subjected to Tuina manipulation for 4 weeks after surgery (thereafter designated as Tuina group). A sham surgery, i.e., with no rod insertion, was conducted on a second group (thereafter designated as sham group), and a third group received CCD surgery but no Tuina manipulation (CCD group). Both sham and CCD groups were handled in the same way as Tuina animals, but with no Tuina manipulation. After CCD surgery, an imaging examination was conducted in CCD and Tuina group to identify the correct position connection between the titanium alloy rod and the nerve roots. The results showed that the titanium rod was successfully fixed to the right foramina of animals in CCD and Tuina group (Figures 2A–E). One rat within the sham group and one rat within the CCD group died during the experiment due to anesthesia and surgery. The remaining rats were subjected to MWT and PWL to assess the effect of Tuina on mechanical and thermal pain sensitivity, respectively. We observed that rats in the sham group presented relatively stable PWL and MWT responses throughout the 4 weeks that followed the surgery. On the contrary, CCD rats presented significantly reduced PWL and MWT responses directly compared to sham group, which decreased to a minimum on D14 (*p* < 0.001) and remained stable until D28 (*p* < 0.001). This indicates that CCD surgery increases pain sensitivity. Finally, animals in the Tuina group exhibited significantly higher MWT and PWL values than CCD animals on 14 (*p* < 0.05), 21 (*p* < 0.01), and D28 (*p* < 0.001) days following the surgery. These results suggest that Tuina reverses CCD impact on pain sensitivity. Altogether, our results show that pain sensitivity created by nerve injury can be restored to its basal level through long-term, non-invasive Tuina intervention (Figures 2F,G).

### 3.2. ALFF maps between the three groups on day 28 following surgery

**Figure 3:**
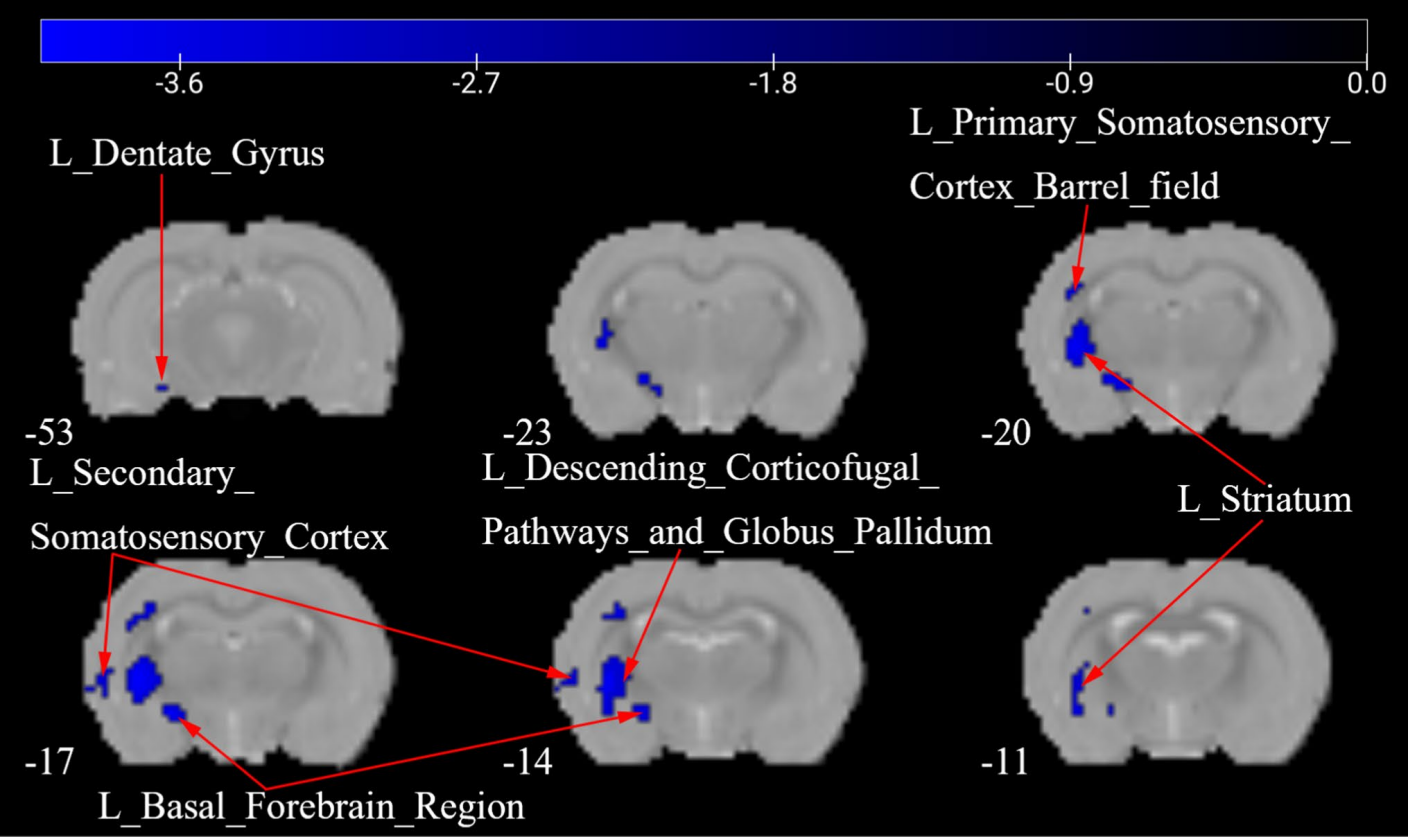
ALFF maps in rats between Tuina group and CCD group 28 days after surgery. The cold tone represents areas where ALFF values of the Tuina group lower than those of the CCD group at 28 days after surgery. The numbers in the figure are the coordinates of the *Z*-axis in standard space. The one-way ANOVA was performed with Bonferroni correction (Voxel-*p* < 0.001, Cluster-*p* < 0.05, Two-tailed). ALFF: the amplitude of low frequency fluctuations; L: Left; R: Right.

Figure 3 presents a one-way ANOVA with Bonferroni correction of ALFF maps between the Tuina and CCD groups at 28 days following surgery. Compared to those of the CCD group, the ALFF values of the Tuina group were significantly elevated 28 days after CCD surgery in the left basal forebrain region, left primary somatosensory cortex barrel field, left dentate gyrus, left secondary somatosensory cortex, left striatum, and left descending corticofugal pathways and globus pallidum (Figure 3; Table 1).

**Table 1:** Brain regions showing ALFF differences between the Tuina group and the CCD group 28 days after surgery.

	Brain regions	No. of voxels	Peak *t*-value	MNI coordinates (mm)		
				x	y	z
Tuina>CCD	L_Basal_Forebrain_Region	29	−4.001	−30	−18	−9
	L_Dentate_Gyrus	2	−3.377	−33	−51	−12
	L_Primary_Somatosensory_ Cortex_Barrel_field	25	−3.962	−39	−15	42
	L_Secondary_Somatosensory_ Cortex	11	−3.591	−63	−15	9
	L_Striatum	88	−4.098	−48	−18	9
	L_Descending_Corticofugal_ Pathways_and_Globus_Pallidum	36	−3.732	−42	−18	9

An one-way ANOVA with Bonferroni correction was used to identify significant voxels. The Voxel-*p* value was set at 0.001, and the Cluster-*p* was set at 0.05. All tests were two-tailed. The corresponding *t*-value was then determined by the *p* values. *x*, *y*, *z*: coordinates of the position of the primary peak in MNI space; ALFF, the amplitude of low frequency fluctuations; MNI, Montreal Neurological Institute; L, Left; R, Right.

**Figure 4:**
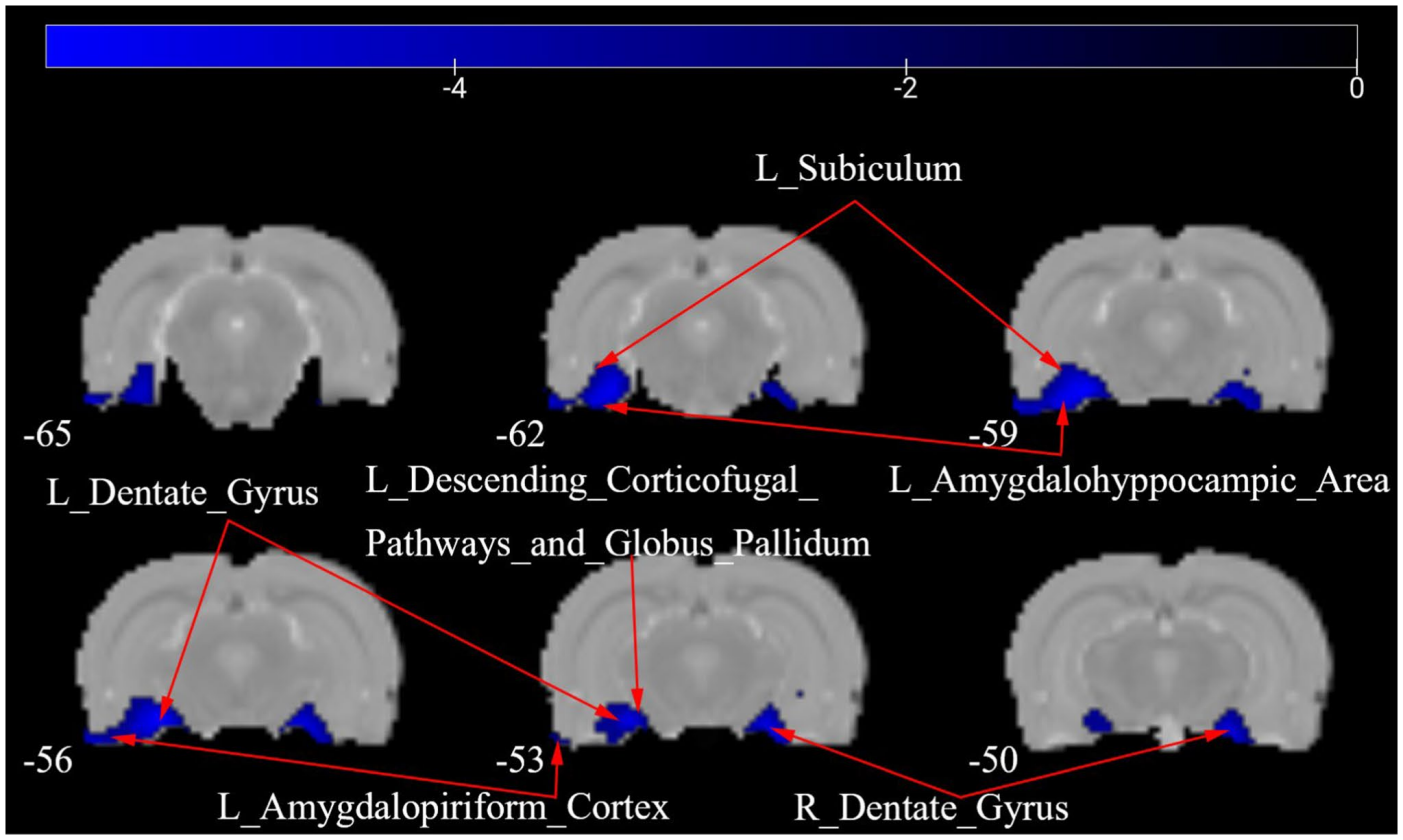
ALFF maps in rats between CCD group and Sham group 28 days after surgery. The cold tone represents areas where ALFF values of the CCD group lower than those of the Sham group at 28 days after surgery. The numbers in the figure are the coordinates of the *Z*-axis in standard space. The one-way ANOVA was performed with Bonferroni correction (Voxel-*p* < 0.001, Cluster-*p* < 0.05, Two-tailed). ALFF, the amplitude of low frequency fluctuations; L, Left; R, Right.

Figure 4 shows a one-way ANOVA with Bonferroni correction of ALFF maps between the CCD and sham groups at 28 days following the surgery. Compared with those of the sham group, the ALFF values of the CCD group were significantly decreased in the left amygdalohyppocampic area, left amygdalopiriform cortex, left subiculum, left descending corticofugal pathways and globus pallidum, and bilateral dentate gyrus (Figure 4; Table 2).

**Table 2:** Brain regions showing ALFF differences between the CCD group and the Sham group 28 days after surgery.

	Brain regions	No. of voxels	Peak *t*-value	MNI coordinates (mm)		
				x	y	z
Sham>CCD	L_Amygdalohyppocampic_Area	15	−5.536	−51	−60	−18
	L_Amygdalopiriform_Cortex	24	−5.606	−48	−60	−15
	L_Dentate_Gyrus	45	−5.550	−33	−57	−12
	R_Dentate_Gyrus	45	−5.437	33	−51	−18
	L_Subiculum	42	−5.548	−36	−54	−12
	L_Descending_Corticofugal_Pathways_and_Globus_Pallidum	1	−3.364	−24	−57	−15

An one-way ANOVA with Bonferroni correction was used to identify significant voxels. The Voxel-*p* value was set at 0.001, and the Cluster-*p* was set at 0.05. All tests were two-tailed. The corresponding *t*-value was then determined by the *p* values. *x*, *y*, *z*: coordinates of the position of the primary peak in MNI space; ALFF, the amplitude of low frequency fluctuations; MNI, Montreal Neurological Institute; L, Left; R, Right.

**Figure 5:**
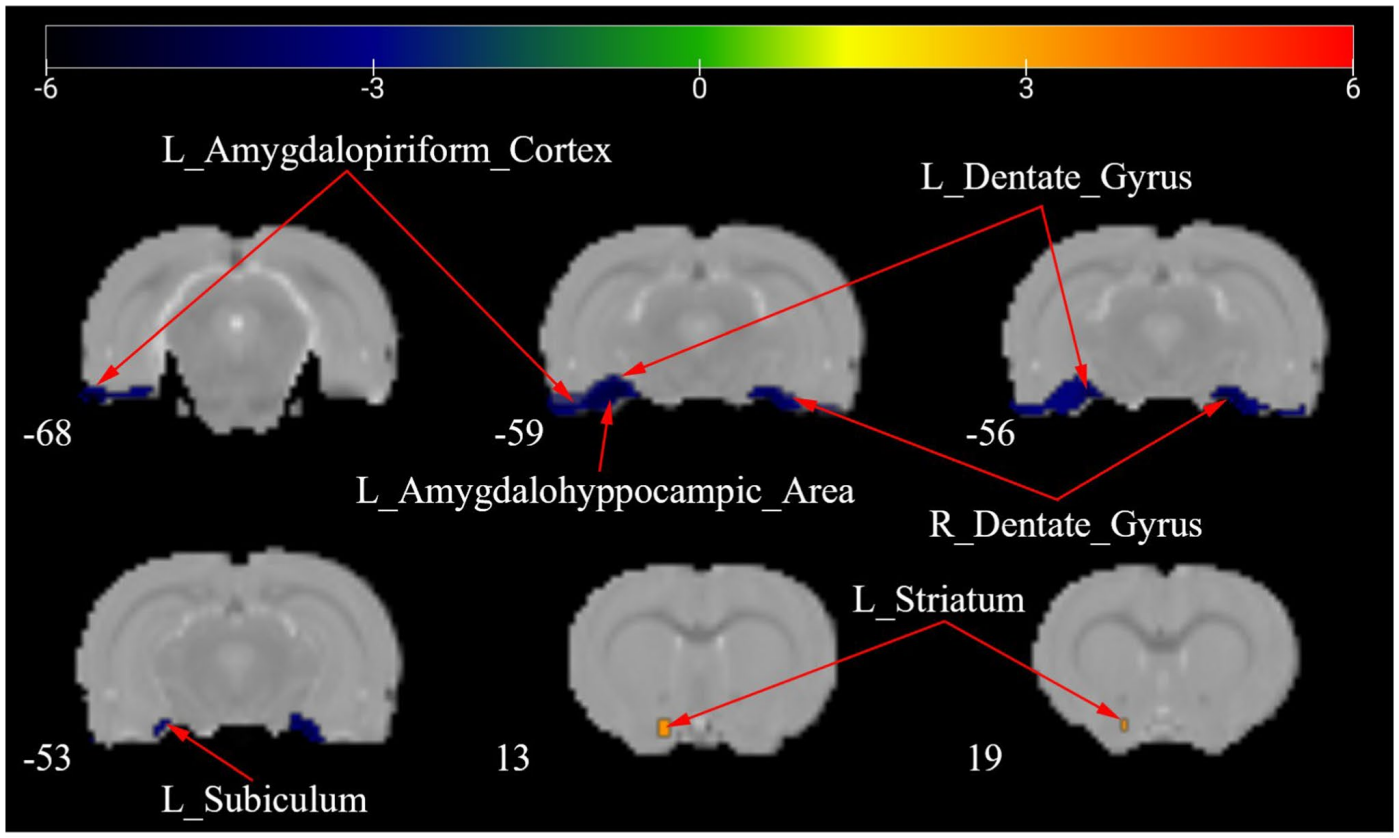
ALFF maps in rats between Tuina group and Sham group 28 days after surgery. The warm tone represents areas where the ALFF values of the Sham group lower than those of the Tuina group, and the cold tone represents areas where ALFF values of the Sham group higher than those of the Tuina group. The numbers in the figure are the coordinates of the *Z*-axis in standard space. The one-way ANOVA was performed with Bonferroni correction (Voxel-*p* < 0.001, Cluster-*p* < 0.05, Two-tailed). ALFF, the amplitude of low frequency fluctuations; L, Left; R, Right.

Figure 5 presents a one-way ANOVA with Bonferroni correction of ALFF maps between the Tuina and sham groups at 28 days following surgery. Compared with those of the sham group, the ALFF values of Tuina group were significantly decreased in the left amygdalohyppocampic area, left amygdalopiriform cortex, left subiculum, and bilateral dentate gyrus. Conversely, ALFF values in the left striatum was significantly increased (Figure 5; Table 3).

**Table 3:** Brain regions showing ALFF differences between the Tuina group and the Sham group 28 days after surgery.

	Brain regions	No. of voxels	Peak *t*-value	MNI coordinates (mm)		
				*x*	*y*	*z*
Tuina>Sham	L_Striatum	8	3.617	−15	15	−18
Sham>Tuina	L_Amygdalohyppocampic_Area	20	−5.654	−51	−60	−18
	L_Amygdalopiriform_Cortex	14	−5.531	−48	−60	−15
	L_Dentate_Gyrus	9	−4.725	−30	−54	−15
	R_Dentate_Gyrus	16	−4.824	33	−51	−18
	L_Subiculum	10	−4.494	−39	−57	−9

An one-way ANOVA with Bonferroni correction was used to identify significant voxels. The Voxel-*p* value was set at 0.001, and the Cluster-*p* was set at 0.05. All tests were two-tailed. The corresponding *t*-value was then determined by the *p* values. *x*, *y*, *z*: coordinates of the position of the primary peak in MNI space; ALFF, the amplitude of low frequency fluctuations; MNI, Montreal Neurological Institute; L, Left; R, Right.

### 3.3. ALFF maps of the Tuina group between days 7 and 28 following surgery

**Figure 6:**
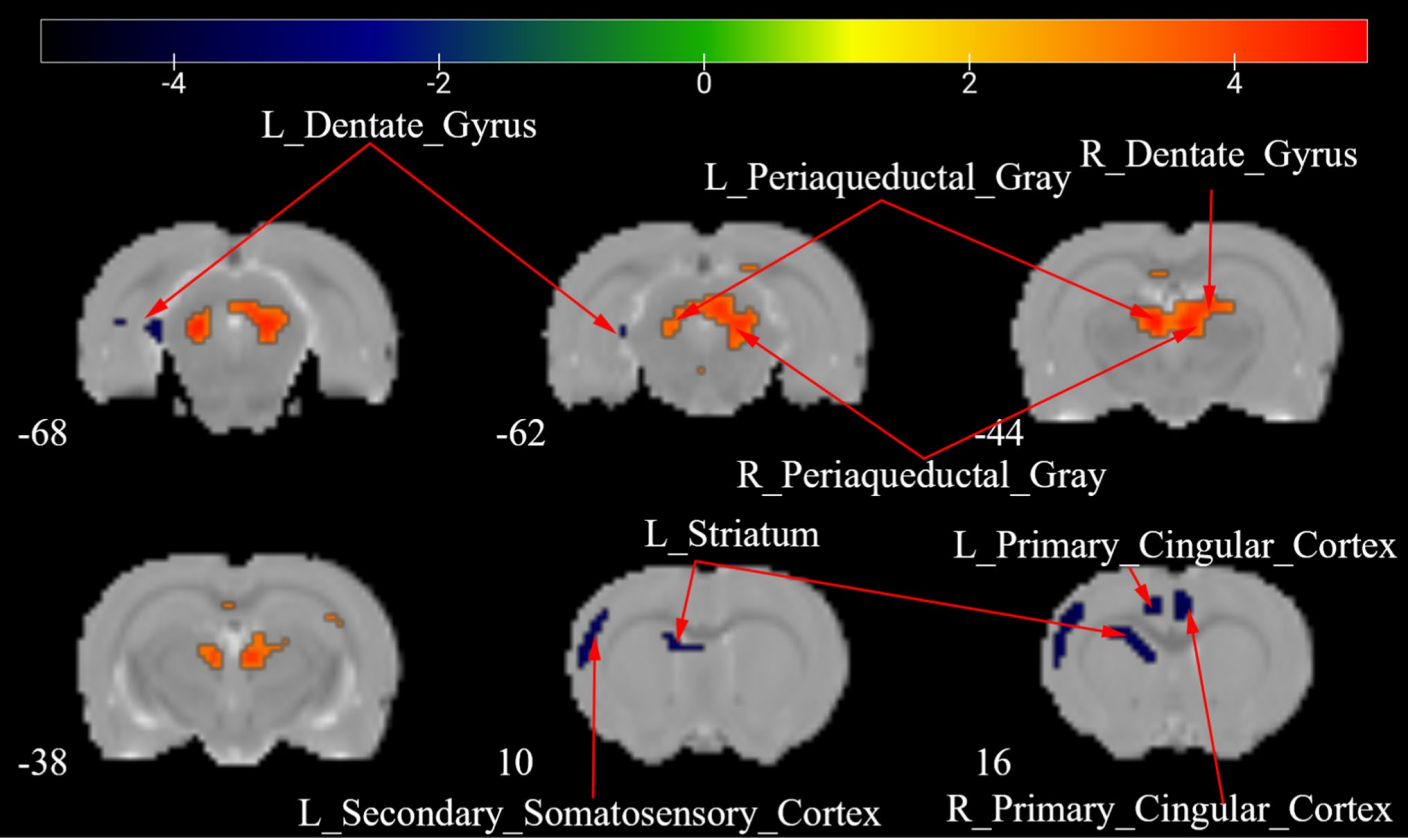
ALFF maps of the Tuina group between 7 days and 28 days after surgery. The warm tone represents areas where the ALFF values at 28 days lower than those at 7 days, and the cold tone represents areas where ALFF values at 28 days higher than those at 7 days. The numbers in the figure are the coordinates of the *Z*-axis in standard space. The one-way ANOVA was performed with Bonferroni correction (Voxel-*p* < 0.001, Cluster-*p* < 0.05, Two-tailed). ALFF: the amplitude of low frequency fluctuations; L, Left; R, Right.

Figure 6 depicts a two-sample t test analysis of the Tuina group ALFF maps between days 7 and 28 following surgery. On the 28th day, the ALFF signals of the left dentate gyrus, left secondary somatosensory cortex, left striatum, and bilateral primary cingulate cortex were significantly elevated contrasted to those on day 7, while signals of the right dentate gyrus and bilateral periaqueductal gray were significantly decreased compared to those on the 7th day (Figure 6; Table 4).

**Table 4:** Brain regions showing ALFF differences of Tuina group between 7 days and 28 days after surgery.

	Brain regions	No. of voxels	Peak *t*-value	MNI coordinates (mm)		
				*x*	*y*	*z*
7d > 28d	R_Dentate_Gyrus	15	4.264	21	−45	33
	L_Periaqueductal_Gray	53	4.773	3	−51	21
	R_Periaqueductal_Gray	94	4.918	6	−51	21
7d < 28d	L_Dentate_Gyrus	8	−3.556	−39	−66	18
	L_Primary_Cingular_Cortex	181	−4.404	−9	30	42
	R_Primary_Cingular_Cortex	192	−4.608	6	30	45
	L_Secondary_Somatosensory_Cortex	5	−3.493	−57	9	18
	L_Striatum	77	−3.606	−24	18	33

An one-way ANOVA with Bonferroni correction was used to identify significant voxels. The Voxel-*p* value was set at 0.001, and the Cluster-*p* was set at 0.05. All tests were two-tailed. The corresponding *t*-value was then determined by the *p* values. *x*, *y*, *z*: coordinates of the position of the primary peak in MNI space; ALFF, the amplitude of low frequency fluctuations; MNI, Montreal Neurological Institute; L, Left; R, Right.

### 3.4. ALFF maps of the CCD group between days 7 and 28 following surgery

**Figure 7:**
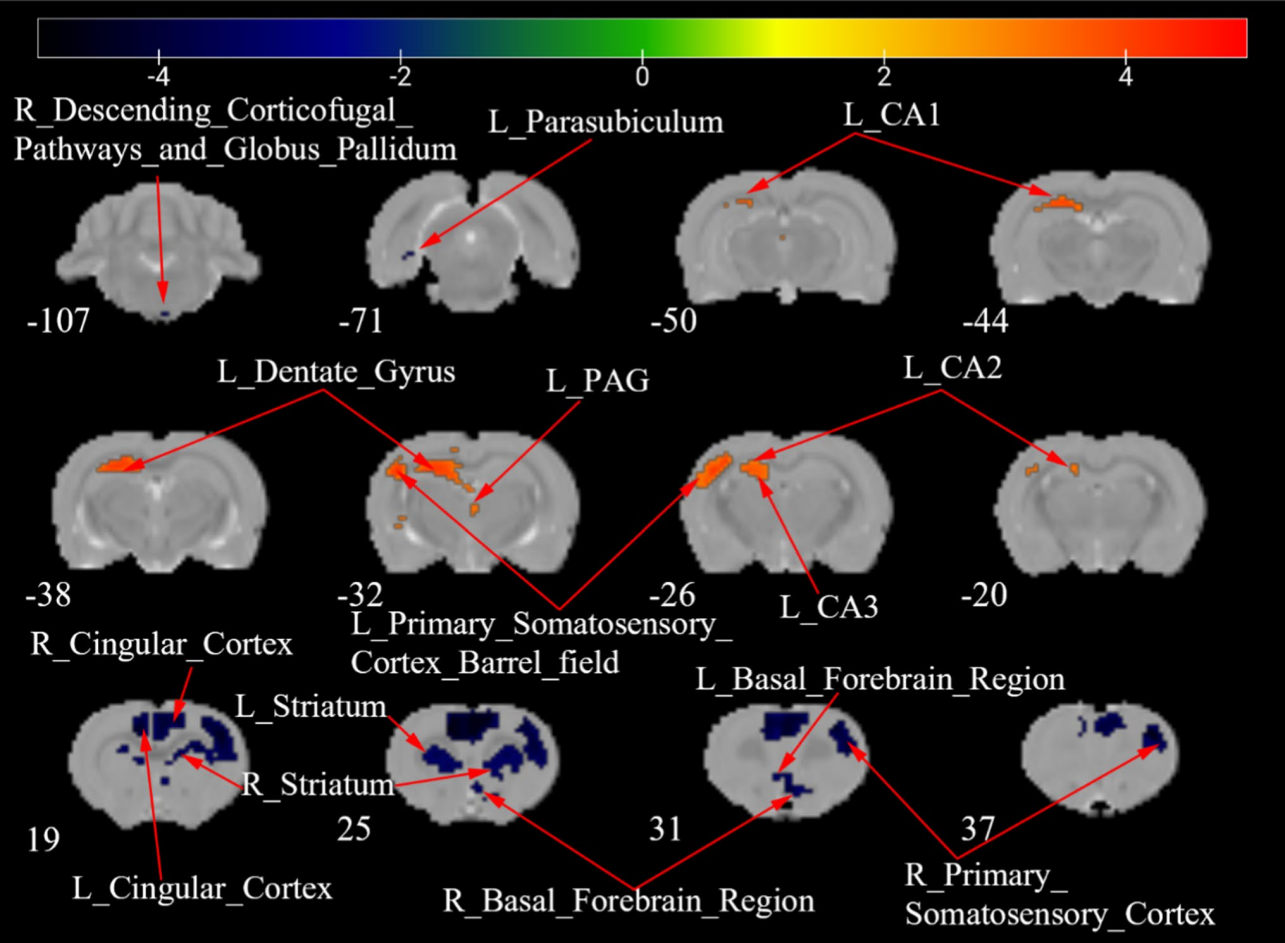
ALFF maps of the CCD group between 7 days and 28 days after surgery. The warm tone represents areas where the ALFF values at 28 days lower than those at 7 days, and the cold tone represents areas where ALFF values at 28 days higher than those at 7 days. The numbers in the figure are the coordinates of the *Z*-axis in standard space. The one-way ANOVA was performed with Bonferroni correction (Voxel-*p* < 0.001, Cluster-*p* < 0.05, Two-tailed). ALFF, the amplitude of low frequency fluctuations; CA, Cornu Ammonis; PAG, Periaqueductal Gray; L, Left; R, Right.

Figure 7 shows a two-sample t test analysis of the CCD group ALFF maps between days 7 and 28 following surgery. On the 28th day, the ALFF signals of the left parasubiculum, right secondary somatosensory cortex, right descending corticofugal pathways, and globus pallidum, bilateral basal forebrain region, bilateral cingulate cortex, and bilateral striatum were significantly elevated contrasted to those on day 7, while signals of the left CA1, left CA2, left CA3, left dentate gyrus, left periaqueductal gray, and left primary somatosensory cortex barrel field were significantly decreased compared to those on day 7 (Figure 7; Table 5).

**Table 5:** Brain regions showing ALFF differences of CCD group between 7 days and 28 days after surgery.

	Brain regions	No. of voxels	Peak *t*-value	MNI coordinates (mm)		
				*x*	*y*	*z*
7d > 28d	L_CA1	185	4.196	−24	−36	51
	L_CA2	24	3.981	−27	−30	42
	L_CA3	10	3.635	−15	−30	36
	L_Dentate_Gyrus	62	4.075	−21	−33	42
	L_Periaqueductal_Gray	10	3.701	3	−48	18
	L_Primary_Somatosensory_Cortex_ Barrel_field	142	4.379	−51	−30	42
7d < 28d	L_Basal_Forebrain_Region	27	−4.076	−6	24	18
	R_Basal_Forebrain_Region	45	−4.433	6	30	0
	L_Parasubiculum	10	−3.477	−42	−72	9
	L_Cingular_Cortex	243	−4.744	−12	27	48
	R_Cingular_Cortex	199	−4.743	12	24	48
	R_Secondary_Somatosensory_Cortex	353	−4.816	42	39	42
	L_Striatum	101	−4.219	−33	24	24
	R_Striatum	170	−4.901	33	24	30
	R_Descending_Corticofugal_Pathways_and_Globus_Pallidum	3	−3.399	9	−108	−36

An one-way ANOVA with Bonferroni correction was used to identify significant voxels. The Voxel-*p* value was set at 0.001, and the Cluster-*p* was set at 0.05. All tests were two-tailed. The corresponding *t*-value was then determined by the *p* values. *x*, *y*, *z*: coordinates of the position of the primary peak in MNI space; ALFF, the amplitude of low frequency fluctuations; MNI, Montreal Neurological Institute; L, Left; R, Right.

### 3.5. Positive association between ALFF values and behavioral tests of the right hindlimb

**Figure 8:**
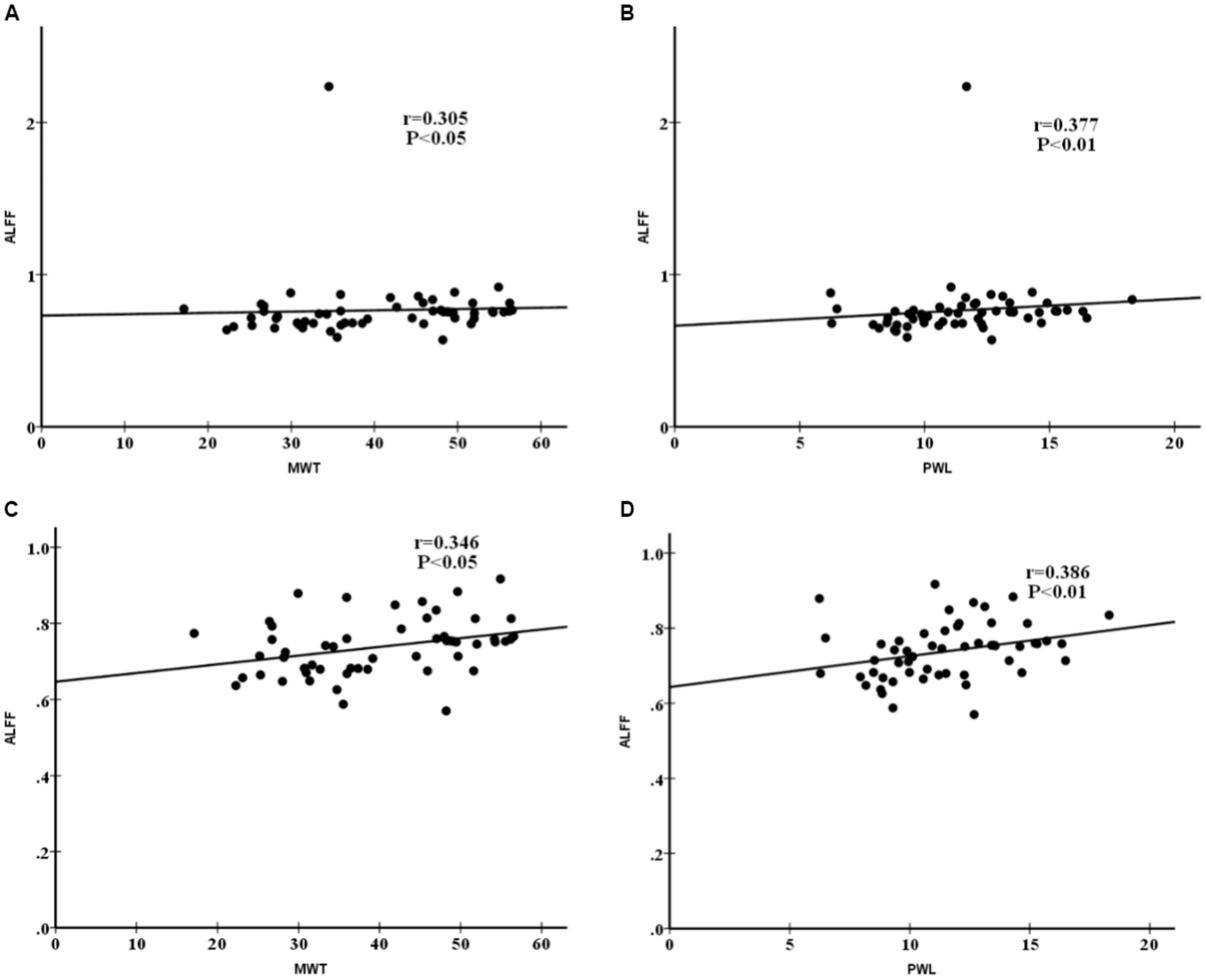
**(A)** Spearman correlation between the ALFF values and MWT. **(B)** Spearman correlation between the ALFF values and PWL. **(C)** Spearman correlation between the ALFF values and MWT after removing outlier. **(D)** Spearman correlation between the ALFF values and PWL after removing outlier. The analysis of all date indicated a positive correlation between the ALFF values of left descending corticofugal pathways and globus pallidum and MWT (r = 0.305, P < 0.05) and PWL (r = 0.377, P < 0.01) in all three groups. After removing a data with a high degree of dispersion, the analysis indicated a positive correlation between the ALFF values of left descending corticofugal pathways and globus pallidum and MWT (r = 0.346, P < 0.05) and PWL (r = 0.386, P < 0.01) in all three groups. The black dots represent the MWT and PWL for rats in three groups at the two time points of 7 and 28 days after CCD. MWT: Mechanical withdrawal threshold; PWL: paw withdrawal thermal latency; ALFF: Amplitude of low frequency fluctuation.

By comparing behavioral test results with ALFF maps, we found an association between ALFF values of the left descending corticofugal pathways and globus pallidum and MWT and PWL. We chose the activation area of the variation among the CCD and sham groups within the ALFF map on day 7 as a mask and obtained ALFF signals of the three groups on days 7 and 28. The ALFF value of the left descending corticofugal pathways and globus pallidum was positively correlated with MWT (*r* = 0.305, *p* < 0.05) and PWL (r = 0.386, *p* < 0.01) (Figures 8A,B) with changed to MWT (*r* = 0.346, *p* < 0.05) and PWL (*r* = 0.377, *p* < 0.01)after removing a discrete point (Figures 8C,D). On day 28, the means ± standard deviation of MWT for the sham, CCD, and Tuina groups were 50.71 ± 6.12, 31.07 ± 6.89 and 43.51 ± 5.31, and the means ± standard deviation of PWL for the sham, CCD, and Tuina groups were 13.25 ± 2.08, 7.88 ± 2.06 and 12.36 ± 2.64, respectively, which indicated a greater level of desirable conduct within the Tuina group than the CCD group.

The analysis of all date indicated a positive correlation between the ALFF values of left descending corticofugal pathways and globus pallidum and MWT (*r* = 0.305, *p* < 0.05) and PWL (*r* = 0.377, *p* < 0.01) in all three groups. After removing a data with a high degree of dispersion, the analysis indicated a positive correlation between the ALFF values of left descending corticofugal pathways and globus pallidum and MWT (*r* = 0.346, *p* < 0.05) and PWL (*r* = 0.386, *p* < 0.01) in all three groups. The black dots represent the MWT and PWL for rats in three groups at the two time points of 7 and 28 days after CCD. MWT: Mechanical withdrawal threshold; PWL: paw withdrawal thermal latency; ALFF: Amplitude of low frequency fluctuation.

## 4. Discussion

Tuina therapy has been utilized for centuries as a therapeutic modality for diverse medical conditions, such as chronic pain, neurodegenerative diseases, cancer, immune diseases, cardiovascular diseases, sleep disorders, mental disorders, skin diseases, pediatric cerebral palsy, and pediatric muscular torticollis (Field, 2016). In China (Yongjun et al., 2020), and increasingly in the population worldwide (Crawford et al., 2016), individuals suffering chronic pain preferentially select non-invasive massage therapy such as Tuina. However, little is known about the analgesic mechanisms of Tuina in the brain. In the present study, the rats of Tuina group underwent CCD surgery and Tuina at the ipsilateral BL40, the rats of CCD group received CCD surgery, while the rats of sham group received sham surgery. All rats received R-fMRI at baseline and 7 days and 28 days following surgery. We observed that Tuina intervention greatly improved the response to both mechanical and thermal pain in NP model. What is more, CCD surgery leads to significant changes in ALFF in multiple brain regions of the contralateral hemisphere while Tuina can partially reverse this change. Altogether, our results indicate that NPP induced by CCD surgery affects the plasticity of the cerebral cortex, and that Tuina alleviate pain behavior by promoting cortical remodeling.

Several randomized controlled investigations have revealed that Tuina can significantly decrease the visual analog scale core, alleviate adverse feelings, and enhance quality of life for patients with neck pain (Lee et al., 2021; Cheng et al., 2022). A multicenter randomized controlled clinical study confirmed that massage can significantly reduce patients’ pain symptoms and dysfunction levels, and is worthy of clinical promotion (Zhou et al., 2022). Another research shows that following a 90-day Tuina treatment, the basic movement, coordination, and obstetrical brachial plexus injury function scores of the individuals with obstetrical brachial plexus injury were significantly enhanced, with the therapy deemed to be effective in all participants (Ma et al., 2018). One single-center, randomized, controlled trial showed that Tuina exhibited more favorable outcomes in terms of pain management, reduction of negative emotions, and improvement of disability over a prolonged duration when compared to celecoxib treatment in patients diagnosed with knee osteoarthritis (Xu et al., 2023). Our present data are consistent with previous studies conducted by our team that had confirmed that Tuina significantly decreased pain behavior in inflammatory and NP rat models (Jiang et al., 2016; Pengfei et al., 2018). Yang et al. (2020) and Yao et al. (2022) determined that Tuina’s effects are predicated on a mechanism that entails a reduction in inflammatory factors, involving IL-1b, IL-6, and TNF-a. Changes in these factors affect modulation of ligand-gated and mechanosensitive ion channels, inhibit red blood cell aggregation and microglia and astrocyte activation, promote blood and lymph circulation, and reduce blood viscosity (Liu et al., 2022). Interestingly, numerous investigations have been conducted to explore alterations in brain plasticity after massage therapy (Sliz et al., 2012). For example, Ouchi et al. (2006) revealed that application of massage therapy in the prone position may lead to a substantial improvement in cerebral blood flow of the parietal cortex. Selective alterations have also been observed in the retro splenial/posterior cingulate region after reflexology massage (Sliz et al., 2012).

fMRI is widely regarded as a crucial neuroimaging modality that enables non-invasive monitoring of brain function (Guerra-Carrillo et al., 2014). Viswanathan and Freeman (2007) documented alterations in tissue oxygen concentration throughout the visual cortex. They proposed that the blood-oxygen-level-dependent (BOLD) fMRI signal predominantly corresponds to neuronal and synaptic activity (Viswanathan and Freeman, 2007). The excitatory/inhibitory balance between synapses is connected to hemodynamics and indirectly affects the BOLD signal intensity; a negative BOLD signal suggests suppression of synapse function (Lauritzen et al., 2012). ALFF values are associated with the spontaneous activity of a particular region of the brain. These values are obtained by utilizing a technique that relies on fast Fourier transformation of the resting-state time series for each voxel (Mohamed et al., 2004). ALFF is purportedly associated with regional cerebral blood flow and is vulnerable to intrinsic or synaptic activity of the brain (Zuo et al., 2010a; Nugent et al., 2015). The resting-state ALFF exhibits a high degree of temporal stability and is frequently employed as a biological indicator for prolonged intervention in different disorders (Küblböck et al., 2014).

NP induces cortical remodeling, as has been determined through analysis of ALFF showing changes in the somatosensory cortex contralateral to the affected hindlimb after sciatic nerve transection (Wu et al., 2018). On the contrary, peripheral afferent malfunction following brachial plexus injury could also cause an increase in ALFF in the ipsilateral sensory cortex (Philip and Frey, 2014). Our study shows that ALFF values of the left amygdalohyppocampic area, left amygdalopiriform cortex, left descending corticofugal pathways and globus pallidum, and left dentate gyrus were significantly decreased after CCD on the right side, indicating that nerve injury reduced spontaneous neuronal activity of the contralateral hemisphere. Moreover, Tuina manipulation restored the neuronal activity in these regions of the brain, as AFLL values in the Tuina group were significantly increased compared to CCD grou*p* values, supporting the contribution of Tuina to sensorimotor functional recovery. Other research has shown that peripheral nerve injury resulted in inhibition of local excitability in the contralateral somatosensory cortex, and the application of Tuina therapy had the potential to restore the somatosensory cortex activity, facilitating the process of information integration, and thereby restoring sensory function (Xing et al., 2021). Furthermore, the cingulate cortex has been hypothesized to perform a crucial function in the processing of information pertaining to the unpleasantness associated with pain (Price, 2000; Wang et al., 2017). We therefore also determined the regulatory effect of Tuina on the cingulate cortex of NP rat model.

In this investigation, we not only analyzed Tuina’s impact on peripheral nerve injury through ALFF signals, but also studied the time-frame during which Tuina affected brain plasticity after CCD surgery. In addition, we conducted a correlation analysis between behavioral test results and ALFF signals of characteristic brain regions, to better understand the regulatory mechanism and target brain regions of Tuina-based analgesia. The result shows that left descending corticofugal pathways and globus pallidum had a positive correlation with MWT and PWL, indicating Tuina alleviate pain behavior by promoting cortical remodeling. CCD-associated paresthesia exhibited persistence while we should place emphasis on the post-therapeutic impact of Tuina as opposed to its real-time effects. We identified alterations in fMRI signal after Tuina intervention that indicated a sustained therapeutic impact.

This study had several limitations. First, the differences between human and rat models limit additional interpretation of the data. Second, we focused on the ALFF of certain brain regions, deliberately omitting the functional connectivity (FC) between various brain regions and the subsequent alterations of brain networks. In future research, we will examine the FC among different regions of the brain and mechanisms of neuromodulation in NP treating with Tuina. Furthermore, neuroinflammation, important in the development of secondary injury, was not evaluated here. Subsequent investigations will include neuroinflammation and its molecular pathways to further explain the analgesic mechanism of Tuina. Finally, physicians often choose manipulation and stimulation intensity according to the condition and tolerance of patients in clinical practice, so the use of a single manipulation and standardized stimulus parameters cannot fully represent the actual therapeutic effect of Tuina.

In conclusion, our study focused on the effect of Tuina intervention on a CCD model and indicated that Tuina relieves pain *via* regulation of neuronal plasticity within both the ipsilateral and contralateral hemispheres. The present research has yielded compelling information that adaptive cerebral plasticity following Tuina therapy persists over a considerable duration of time (25 days). These outcomes provide further insight into the alterations in cortical plasticity resulting from the prolonged impact of Tuina on an NP model.

## Data availability statement

The original contributions presented in the study are included in the article/supplementary material, further inquiries can be directed to the corresponding author.

## Ethics statement

The animal study was reviewed and approved by the Institutional Animal Care Committee of Yueyang Hospital of Integrated Traditional Chinese and Western Medicine, the Shanghai University of Traditional Chinese Medicine (YYLAC-2022-148-5).

## Author contributions

ZW and MF conceived and designed the experiments. ZW, GG, and QZ conducted manuscript writing. ZW and YZ carried out the fMRI image acquisition. YL, GG, and YZ conducted data analysis. TH helped with the behavior test experiments. LK conducted data collection and helped manuscript writing. All authors had read and approved the final version of the manuscript.

## Funding

This work was supported by the National Natural Science Foundation of China (82205302, 82030121,and 82105042), Shanghai Sailing Program (20YF1450900), Science Foundation of Yueyang Hospital of Integrated Traditional Chinese and Western Medicine (2021yygq03), and Traditional Chinese Medicine Research Project of Shanghai Municipal Health Commission (2022QN059).

## Conflict of interest

The authors declare that the research was conducted in the absence of any commercial or financial relationships that could be construed as a potential conflict of interest.

## Publisher’s note

All claims expressed in this article are solely those of the authors and do not necessarily represent those of their affiliated organizations, or those of the publisher, the editors and the reviewers. Any product that may be evaluated in this article, or claim that may be made by its manufacturer, is not guaranteed or endorsed by the publisher.

## Footnotes

^1^ https://www.fil.ion.ucl.ac.uk/spm/software/spm12
